# Bilevel optimization for automated machine learning: a new perspective on framework and algorithm

**DOI:** 10.1093/nsr/nwad292

**Published:** 2023-11-21

**Authors:** Risheng Liu, Zhouchen Lin

**Affiliations:** School of Software Technology, Dalian University of Technology, China; School of Intelligence Science and Technology, Peking University, China

**Keywords:** automated machine learning, bilevel optimization, meta feature learning, neural architecture search, hyperparameter optimization

## Abstract

Formulating the methodology of machine learning by bilevel optimization techniques provides a new perspective to understand and solve automated machine learning problems.

Machine learning (ML) has witnessed an unprecedented evolution in recent years, becoming a key driver of building artificial intelligence systems. With cutting-edge technologies such as AlphaGo [1] and ChatGPT [2], the power and versatility of ML have been demonstrated across diverse applications. However, designing effective ML solutions in real-world application scenarios can be challenging and time-consuming, thus paving the way for the emergence of automated machine learning (AutoML). AutoML refers to a set of technologies that streamline the entire process of applying ML to complex problems by automating many of the traditionally manual tasks involved in ML. By doing so, AutoML enables the generation of more powerful ML solutions and extends their scope of applicability [3].

In this perspective, we investigate the intrinsic mechanisms and (re)formulate these different AutoML tasks from a unified optimization perspective. Figure 1(a) illustrates how we can view the process of AutoML as addressing the three key issues of ML tasks: how to extract the learning feature, how to construct the learning model and how to design the learning strategy. These three issues correspond to the main techniques of AutoML, namely, meta feature learning (MFL), neural architecture search (NAS) and hyperparameter optimization (HO), respectively.

**Figure 1.**
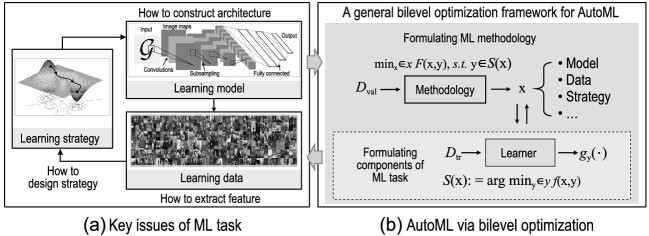
(a) Illustration of key issues of ML task. The network structure is plotted based on AlexNet (https://en.wikipedia.org/wiki/AlexNet), the set of images are sampled from COCO dataset (https://cocodataset.org/), and the energy surface is generated using the peaks function from MATLAB. (b) Formulation of AutoML paradigm from the perspective of bilevel optimization.

In essence, MFL enables us to automatically extract relevant features for unseen new tasks [4], NAS helps us to design effective neural network architectures [5] and HO assists us in finding optimal hyperparameters for the model [6]. By automating these key aspects of the ML pipeline, AutoML frees up valuable time and resources for practitioners to focus on other critical tasks. Overall, the techniques of MFL, NAS and HO play crucial roles in enabling AutoML to efficiently and effectively handle ML tasks. Very recently, Shu *et al.* [7] provided a simulating learning methodology (SLeM), a general paradigm with solid theoretical guarantees for predicting proper hyperparameter configurations for various AutoML applications.

Bilevel optimization (BLO) refers to a category of mathematical tools for hierarchical optimization with two levels of problems: an upper-level problem and a lower-level problem [8]. In the context of AutoML, we can actually utilize BLO to uniformly formulate different kinds of AutoML tasks, such as MFL, NAS and HO.

Specifically, we can observe in Fig. 1(b) that in the upper-level problem, the goal is to find the best ‘methodology’ that optimizes the performance of the machine learning model (e.g. meta features, network architectures and tuned hyperparameters). This can be formulated as an optimization problem where the objective function *F* is the performance of the model on a validation set $\mathcal {D}_{\mathtt {val}}$, and the variables $\mathbf {x}\in \mathcal {X}$ are some ‘meta-parameters’ (e.g. corresponding to feature extraction, the network architecture and the learning strategy). The constraints can include factors such as computational resources and time limits. In the lower-level problem, the objective is to optimize the machine learning model itself, *g*, given the meta-parameters chosen in the upper-level problem. It can be formulated as an optimization problem where the objective *f* is the performance of the model on a training set $\mathcal {D}_{\mathtt {tr}}$, and the variables $\mathbf {y}\in \mathcal {Y}$ are parameters of the learning model. Therefore, BLO provides a powerful framework for AutoML, enabling automatic selection and optimization of ML models, and making it possible to build high-performing models with minimal manual intervention.

In the field of ML/AutoML, there has been a recent surge in developing gradient-based techniques for BLOs. Two main categories of such algorithms have emerged in recent years: gradient with explicit differentiation and implicit differentiation. The key difference between these two categories lies in the way they compute the coupled gradients for BLOs. Very recently, a series of single-loop techniques have also been proposed to reduce the complexity of computing the coupled gradients [9]. For further information on these recent developments in gradient-based BLOs, see [8].

Despite the substantial amount of literature in the field, fundamental issues still exist in the current algorithms. One of the major challenges is that many of these studies, including both algorithmic design and theoretical investigations, heavily rely on restrictive conditions such as the lower-level singleton and convexity. Although there have been a few attempts to address this issue [10], it remains a significant obstacle. Another challenge is the difficulty in providing strict convergence analysis on the approximated schemes used in practical applications, without exact calculation of coupled gradients [5].

Ultimately, the best approach to solving a specific BLO problem will depend on the problem structure, the complexity of the objective functions and constraints, and the computational resources available. Thus, it is important to carefully consider the problem formulation and choose an appropriate algorithm or combination of algorithms to efficiently and accurately solve the problem.

Last but not least, it is necessary to provide some discussions on the challenging and promising directions of BLOs for AutoML in the future.

- *Computational acceleration.* As the size and complexity of datasets/tasks continue to increase, there is a pressing need for acceleration techniques to BLO algorithms in extremely large-scale and high-dimensional AutoML applications. This includes designing AutoML algorithms that can efficiently search through a large space of architectures, as well as extracting high-dimensional features and optimizing complex training processes. One promising direction is to explore parallel/distributed computing techniques to accelerate the training and evaluation of models.
- *Theoretical breakthrough.* Existing theories of gradient-based BLOs mostly rely on strong assumptions (e.g. lower-level singleton and convexity [8]), which limit their applications in real-world scenarios. Thus, it is necessary to establish new analyzing tool that can systematically analyze the properties of the BLO landscape and design efficient algorithms for challenging AutoML tasks (e.g. tackling non-convex and discrete learning).
- *Optimization-inspired AutoML.* Currently, BLOs are predominantly recognized as solution strategies for practical AutoML applications. Indeed, by delving into the underlying structure of the AutoML paradigm from the perspective of BLO, we can better capture the complex dependencies between different components of the model and thus have the ability to design more efficient and effective AutoML strategies. For example, integrating the SLeM mechanism and prompt learning techniques within the BLO framework to improve the generalization capability of fundamental vision-language models.
